# ‘Ablate and pace’ reduces mortality in heart failure patients with atrial fibrillation: an updated meta-analysis

**DOI:** 10.1093/ehjopen/oeag020

**Published:** 2026-02-18

**Authors:** Christian Lewinter, John G F Cleland, Eslem Sögütlü, Torsten Holm Nielsen, Hannes Hagström, Lars Køber, Martin LeWinter, Robert Edfors, Cecilia Linde, Frieder Braunschweig

**Affiliations:** Heart Centre, Karolinska University Hospital, Eugeniavägen 3, SE-171 76 Stockholm, Sweden; School of Cardiovascular and Metabolic Health, University of Glasgow, University Avenue, G12 8TA Glasgow, UK; Karolinska Institutet, Nobels väg 6, SE-171 77 Stockholm, Sweden; School of Cardiovascular and Metabolic Health, University of Glasgow, University Avenue, G12 8TA Glasgow, UK; Karolinska Institutet, Nobels väg 6, SE-171 77 Stockholm, Sweden; Danish Medicines Agency, Axel Heides Gade 1, DK-2300 Copenhagen S, Denmark; Department of Haematology, Zealand University Hospital, Sygehusvej 10, DK-4000 Roskilde, Denmark; Karolinska Institutet, Nobels väg 6, SE-171 77 Stockholm, Sweden; Heart Centre, Rigshospitalet, Copenhagen University Hospital, Blegdamsvej 9, DK-2100 Copenhagen Ø, Denmark; Division of Cardiovascular Medicine, White River Junction Veterans Affairs Medical Center, 215 North Main Street, White River Junction, VT 05009, USA; Karolinska Institutet, Nobels väg 6, SE-171 77 Stockholm, Sweden; Heart Centre, Karolinska University Hospital, Eugeniavägen 3, SE-171 76 Stockholm, Sweden; Karolinska Institutet, Nobels väg 6, SE-171 77 Stockholm, Sweden; Heart Centre, Karolinska University Hospital, Eugeniavägen 3, SE-171 76 Stockholm, Sweden; Karolinska Institutet, Nobels väg 6, SE-171 77 Stockholm, Sweden

**Keywords:** Ablate and pace, Atrial fibrillation, Heart failure, Meta-analysis, All-cause mortality, Left ventricular ejection fraction

## Abstract

**Aims:**

We compared the effects of ‘ablate and pace’ to pharmacological therapy on mortality and left ventricular ejection fraction (LVEF) in patients with atrial fibrillation (AF), with or without heart failure (HF).

**Methods and results:**

Articles were identified by searching PubMed, Central, and Embase until 30 June 2024. Inclusion criteria encompassed observational and randomized controlled trials (RCTs) comparing ‘ablate and pace' with pharmacological therapy and investigating outcomes of mortality and LVEF in patients with AF. An exclusion criterion was lack of a parallel study design. The primary outcomes were all-cause mortality and the mean difference (MD) in LVEF. Endpoints were assessed through meta-analyses computing relative risks (RRs) and MDs. The clinical diagnosis of HF was used to distinguish between patients with and without HF. Initially, 3837 studies were identified, of which 24 (*n* = 4292 patients) fulfilled the inclusion criteria, including 17 (*n* = 3261 patients) that focused on HF. Follow-up time varied from 3 to 96 months. Only in HF patients, ‘ablate and pace' reduced mortality significantly with a risk reduction of 36% [RR, 0.64; 95% confidence interval (CI), 0.49–0.85; *P* < 0.01; *n* = 10] as compared with pharmacological therapy. Except for two studies, cardiac resynchronization therapy (CRT) was the chosen pace mode. The mortality reduction was independent of study design: RCTs (RR, 0.41; 95% CI, 0.18–0.94; *P* = 0.04; *n* = 2) and observational studies (RR, 0.70; 95% CI, 0.55–0.90; *P* = 0.01; *n* = 8). ‘Ablate and pace’ and pharmacological therapy were similar for the LVEF outcome (MD, 1.1; 95% CI, −1.6–3.8; *P* = 0.39; *n* = 16), which was independent of both HF and study designs (results not shown).

**Conclusion:**

‘Ablate and CRT’ reduced mortality in HF patients as compared with pharmacological therapy, which was supported by statistical associations in observational studies. A single RCT corroborated the finding.

## Graphical Abstract

**Graphical Abstract:**
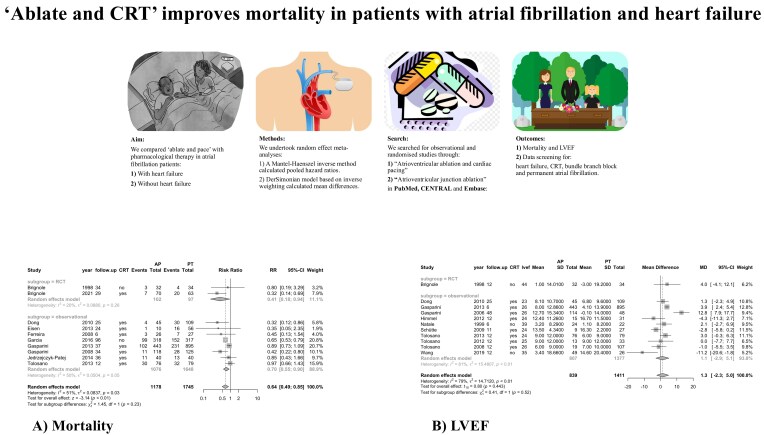

## Introduction

When to set in with ‘ablate and pace’—atrioventricular junction ablation and pacemaker—and which patients with atrial fibrillation (AF) will benefit most are unidentified. The need for better symptom control in medically refractory AF supports an ‘ablate and pace’ strategy.^1^ ‘Ablate and pace’ allows for full-time rate control and ventricular rhythm regularization. The most recent ablation for paroxysmal atrial fibrillation (APAF) randomized controlled trial (RCT) enrolled 133 heart failure patients with symptomatic, permanent AF without bundle branch block (BBB). The trial demonstrated that ‘ablate and cardiac resynchronization therapy (CRT)’ reduces mortality compared to pharmacological therapy.^2^ Yet, the influence of CRT, permanent AF duration, and symptoms on ‘ablate and pace’ outcomes remains uncertain. Mortality and left ventricular ejection fraction (LVEF) outcomes may illuminate such uncertainties since they report hard clinical and physiological endpoints, respectively. Here, we present a comparison of ‘ablate and pace’ vs. pharmacological therapy in a systematic review and meta-analysis of randomized and observational studies of AF patients. Our predefined outcomes are (i) mortality and (ii) LVEF and will include heart failure status too. Secondly, we will assess how CRT, permanent AF duration, and BBB influence on the outcomes.

## Methods

We used the Preferred Reporting Items for Systematic Reviews and Meta-Analyses and Meta-analysis Of Observational Studies in Epidemiology checklists to report on observational studies and RCTs, respectively.^3,4^ We prespecified a protocol in PROSPERO (CRD42022344232).

### Search methods

Broad search terms—‘atrioventricular ablation’ OR ‘atrioventricular junction ablation’ AND ‘pacing’ OR pacemaker’—were separately performed in PubMed, Central, and Embase with a search cut-off date of 30 June 2024. By the same search terms, we screened previous meta-analyses and systematic reviews, guidelines, and statements from cardiovascular society meetings. Abstracts from congresses were not assessed due to their less detailed descriptions of research methods. Titles and abstracts were read first and if found relevant, the full article was reviewed. The search process was undertaken independently by two reviewers (C.L. and E.S.).

### Study and data collection

Observational studies and randomized trials were included for analysis if they conducted a parallel design comparing an intervention group of ‘ablate and pace’ with a control group having pharmacological therapy. An ethics committee approval was not applied due to non-disclosure of patients’ identity. Studies, including patients planned for pulmonary vein isolation (PVI) were excluded. Endpoints at study follow-up were (i) all-cause mortality and (ii) LVEF (%). Left ventricular ejection fraction was enumerated as the mean difference (MD) of the value measured at the beginning and at follow-up of each study. Left ventricular ejection fraction measurement included only echocardiography. Study details, including participant numbers and observation length, were collected based on the majority principle when categorized as binary. Baseline characteristics of age, gender, LVEF, BBB, heart failure status, symptoms, permanent AF, pacemaker (PM) subtype, and history of ischaemic heart disease (IHD) were also collected. Finally, safety, including side effects of prescribed atrioventricular blocking drugs and PM complications, were tabulated.

Study selection was made independently by two reviewers (C.L. and E.S.). In case of disagreement between the reviewers that could not be resolved, a third author (F.B.) made the decision.

### Outcomes

Our primary outcomes were all-cause mortality and LVEF (%), analysed separately for patients with and without heart failure. For this meta-analysis, we included studies enrolling both populations. In studies with heart failure, patients were required to have symptomatic heart failure with reduced ejection fraction, defined as New York Heart Association (NYHA) Class II–IV symptoms despite guideline-directed medical therapy, an LVEF < 40% confirmed by echocardiography, and evidence of structural heart disease and/or ventricular conduction delay when applicable. Minor deviations were accepted if the study population was clearly described as having moderate to severe systolic heart failure based on clinical assessment and imaging. Studies without heart failure were also included to enable comparison of outcomes between these groups.

### Statistics

Discrete data were presented as percentages (%), and continuous data were reported as means and standard deviations (SDs). The 95% confidence intervals (CIs) were defined as the mean ± 1.96 × standard error (SE) in a normal distribution. The SD was the SE × √*n* where *n* was the number of study participants. All meta-analyses were undertaken through a random effect model due to presumption of moderate to high heterogeneity in observational studies and RCTs for inclusion. An estimate of heterogeneity among studies was reported as the *I*^2^ for each analysis.

A Mantel–Haenszel inverse method calculated pooled relative risks (RRs) for all-cause mortality. A DerSimonian model based on inverse weighting calculated the LVEF MD between groups as the change from baseline to the end of the follow-up time. Test statistics and 95% CIs were adjusted according to the Hartung and Knapp method.^5^

*P* < 0.05 were considered significant.

The Meta programme in R statistics was applied for the data calculations.^6^

Details of the sensitivity analysis and risk of bias can be found in the Supplementary material.

## Results

### Search result

Twenty-four studies were selected for the final meta-analysis from the initial data search (Supplementary material).^2,7–29^ Of these, 17 studies included heart failure patients (*Table 1*). We did not find complementary studies from previous meta-analyses. No additional studies were found when newest guidelines and cardiovascular society meetings were investigated. Endpoints of mortality were measured in 14 studies and the LVEF MDs in 16, respectively (six studies measured both). Randomized controlled trials made up four of the studies.

**Table 1:** Baseline characteristics

Study	Year	RCT	No.	Follow-up	Outcome	Women (%)	Age	LVEF (%)	CHF	IHD (%)	PM	AVBD	Permanent	QOL	Symptoms	BBB
Brignole	1997	Yes	43	6	LVEF	53	65	59	No	26	DDD	CCB	No	Yes	Yes	No
Brignole	1998	Yes	66	34	Both	53	72	44	Yes	38	VVI	CCB	Yes	Yes	Yes	No
Brignole	2021	Yes	133	29	Both	47	73	41	Yes	31	CRT	BB	Yes	No	Yes	No
Dong	2010	No	154	25	Both	14	71	23	Yes	58	CRT	BB	na	No	No	Yes
Eisen	2013	No	66	24	Dead	21	70	24	Yes	72	CRT	BB	No	No	Yes	Yes
Ferreira^a^	2008	No	53	6	Dead	6	69	25	Yes	53	CRT	BB	na	No	Yes	Yes
Garcia^a^	2016	No	635	96	Dead	49	75	44	Yes	36	VVI	BB	No	No	Yes	No
Gasparini	2006	No	162	48	LVEF	14	66	26	Yes	37	CRT	BB	Yes	No	Yes	Yes
Gasparini	2008	No	243	34	Dead	18	66	26	Yes	40	CRT	BB	Yes	No	Yes	Yes
Gasparini	2013	No	1338	37	Both	15	69	26	Yes	37	CRT	BB	Yes	No	Yes	Yes
Himmel	2012	No	46	12	LVEF	14	69	24	Yes	63	CRT	BB	Yes	No	Yes	Yes
Jedrzejczyk-Patej	2014	No	80	36	Dead	9	60	24	Yes	46	CRT	BB	No	No	Yes	Yes
Lim	2007	No	33	65	Both	24	74	58	No	42	VVI	D	Yes	Yes	Yes	Yes
Nagamoto	2011	No	28	6	Dead	57	75	66	No	29	VVI	na	No	Yes	Yes	No
Natale	1999	No	46	6	LVEF	33	69	39	Yes	24	VVI	CCB	Yes	Yes	Yes	No
Ozcan^a^	2001	No	579	36	Dead	43	68	48	No	41	DDD	D	No	No	Yes	No
Schütte	2009	No	36	11	LVEF	14	70	24	Yes	68	CRT	BB	Yes	No	Yes	Yes
Tolosano	2008	No	126	12	LVEF	19	69	26	Yes	31	CRT	D	Yes	Yes	Yes	Yes
Tolosano	2012	No	46	12	LVEF	29	68	25	Yes	32	CRT	BB	Yes	Yes	Yes	Yes
Tolosano	2013	No	155	12	Both	19	69	25	Yes	36	CRT	BB	Yes	Yes	Yes	Yes
Ueng	2001	No	47	12	LVEF	30	66	45	No	na	VVI	na	Yes	Yes	Yes	No
Wang^a^	2019	No	75	12	LVEF	25	68	36	Yes	18	HPSP	BB	Yes	No	Yes	No
Weerasooriya	2003	Yes	99	12	Both	30	68	56	No	40	VVI	na	Yes	Yes	Yes	No
Wong	1996	No	18	3	LVEF	50	67	45	No	na	VVI	yes	No	No	na	No

Detailed distributions are provided in the Supplementary material.

RCT, randomized controlled trials; No., number of patients; follow-up is expressed in months; age is expressed in years; LVEF, left ventricular ejection fraction; IHD, ischaemic heart disease; PM, pacemaker; VVI, right single-chamber lead; CRT, cardiac resynchronization therapy; DDD, right double-chamber lead; HPSP, His–Purkinje system pacing; AVBD, atrioventricular blocking drug; CCB, calcium channel blocker; BB, beta-blocker; D, digoxin; na, non-applicable; Permanent, permanent atrial fibrillation; QOL, quality of life outcomes; BBB, bundle branch block; CHF, chronic heart failure.

^a^Studies marked with an asterisk included heterogeneous samples comprising both CRT and DDD(R)/VVI patients.

### Baseline characteristics

A total of 4292 AF patients were included in the meta-analysis out of which 3261 had heart failure. The mean follow-up time was 6–96 months for patients with heart failure (without heart failure, 3–65 months). The mean age of included studies varied between 60 and 75 years in heart failure patients as compared with 65–75 years in patients without heart failure (*Table 1*). The proportion of women with heart failure was 6–49% (without heart failure, 24–57%). Among studies with available data (*n* = 22), permanent AF was reported in 12 studies enrolling patients with heart failure and in three studies enrolling patients without heart failure. The mean baseline LVEF ranged from 23 to 44% in heart failure patients (without heart failure, 41 to 66%). Ischaemic heart disease was reported in 18–72% among heart failure patients (without heart failure, 26–42%). In heart failure patients, CRT was reported in all but four studies (84%) (without heart failure, zero studies).

### Primary outcomes

#### Mortality

**Figure 1:**
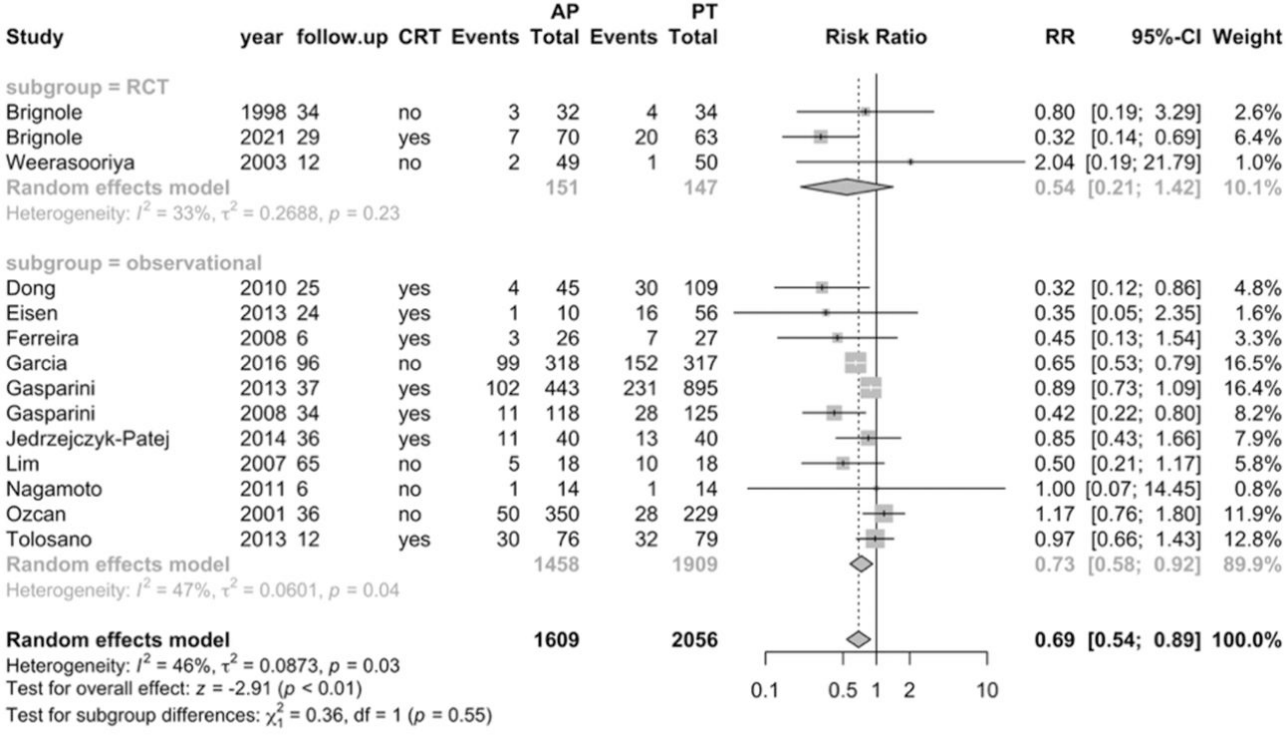
Forest plot of mortality for all included studies, comparing ‘ablate and pace’ ( *n* = 1609; 63.8% with cardiac resynchronization therapy) vs. pharmacological therapy (*n* = 2056). Hazard ratios were presented as relative risk. RCT, randomized controlled trial; CRT, cardiac resynchronization therapy; follow-up, months; AP, ablate and pace; PT, pharmacological therapy; relative risk (RR).

Mortality outcomes were reported for a total of 3665 AF patients across 14 studies, with a mean follow-up ranging from 6 to 96 months. The forest plot (*Figure 1*) summarizes mortality data comparing ‘ablate and pace’ (*n* = 1609) with pharmacological therapy (*n* = 2056). Overall, 329 (20%) of patients in the ‘ablate and pace’ group died, compared with 573 (28%) in the pharmacological therapy group. Pooled analysis demonstrated that ‘ablate and pace’ was associated with a significantly lower RR of mortality (RR, 0.69; 95% CI, 0.54–0.89; *I*² = 46%; *P* < 0.01), particularly among patients with heart failure. Cardiac resynchronization therapy was used in 826 (52%) of patients in the ablate and pace group.

**Figure 2:**
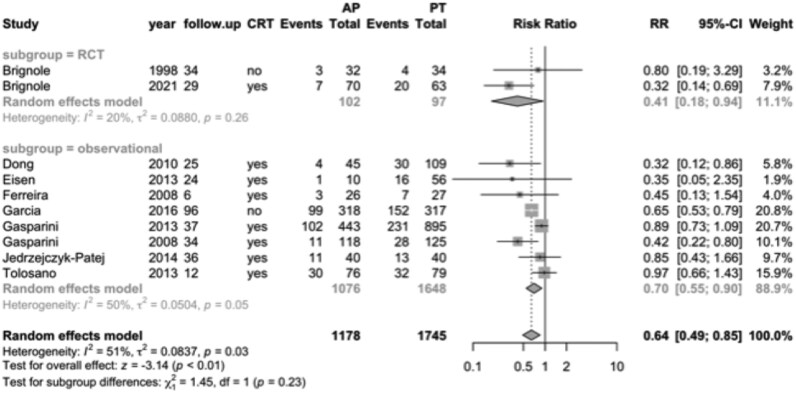
Forest plot of mortality for the heart failure population, comparing ‘ablate and pace’ (*n* = 1178) vs. pharmacological therapy (*n* = 1745). RCT, randomized controlled trial; CRT, cardiac resynchronization therapy; follow-up, months; RR, relative risk; AP, ablate and pace; PT, pharmacological therapy.

In the subset of eight observational studies involving heart failure patients, mortality was 22% in the ‘ablate and pace’ group (*n* = 1076) compared to 27% in the pharmacological therapy group (*n* = 1648), yielding a RR of 0.70 (95% CI, 0.55–0.90; *Figure 2*). Two RCTs enrolling a total of 199 patients with heart failure reported mortality rates of 10% in the ‘ablate and pace’ group vs. 25% in the pharmacological therapy group (RR, 0.41; 95% CI, 0.18–0.94).

**Figure 3:**
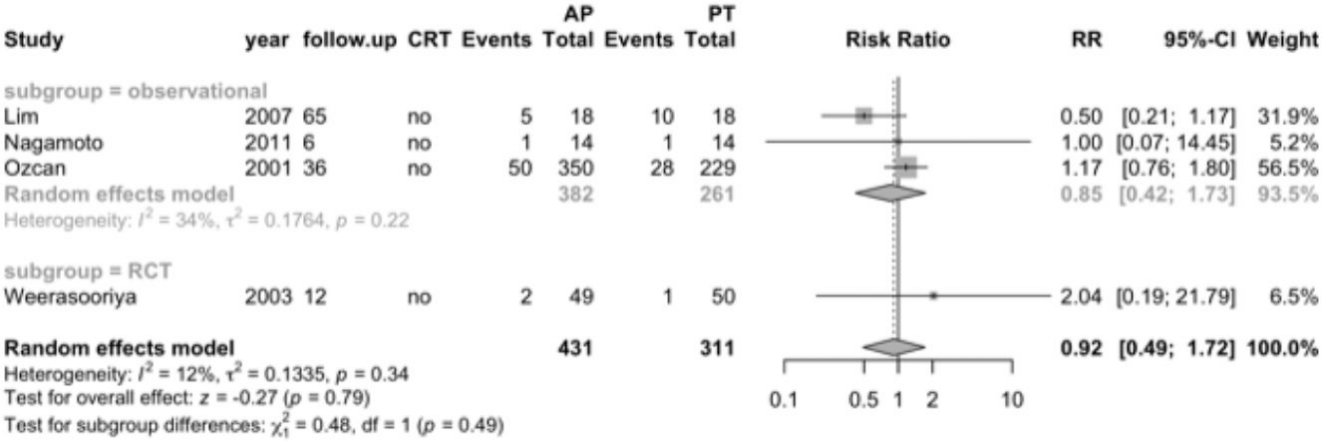
Forest plot of mortality for the population without heart failure, comparing ‘ablate and pace’ (*n* = 431) vs. pharmacological therapy (*n* = 311). RCT, randomized controlled trial; CRT, cardiac resynchronization therapy; follow-up, months; RR, relative risk; AP, ablate and pace; PT, pharmacological therapy.

Among patients without heart failure, four observational studies included 382 patients treated with ‘ablate and pace’ and 261 patients treated with pharmacological therapy. In the pooled analysis, mortality occurred in 13% of both groups. There was no significant difference in mortality between the two treatment strategies (RR 0.92; 95% CI, 0.49–1.72; *Figure 3*). Similarly, a single RCT involving 99 patients without heart failure reported no significant difference in mortality between the ‘ablate and pace’ group (4%) and the pharmacological therapy group (2%) (RR 2.04; 95% CI, 0.19–21.8).

#### Left ventricular ejection fraction

**Figure 4:**
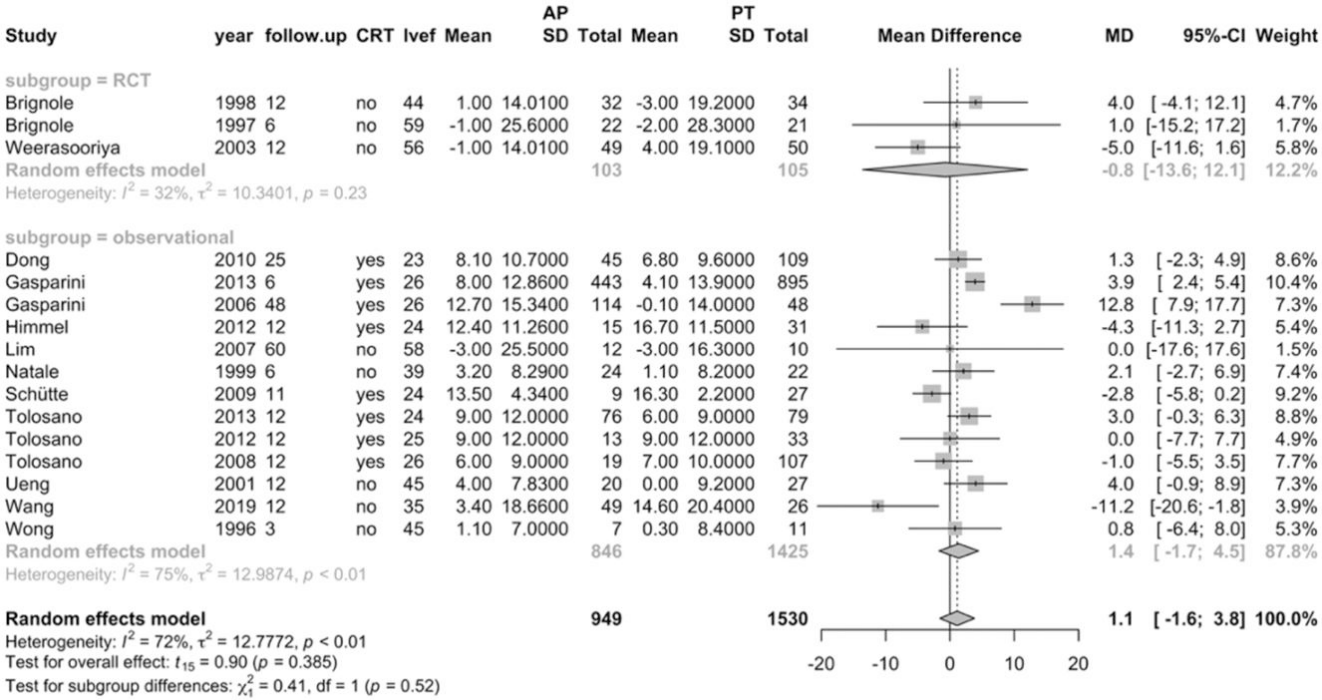
Forest plot of mean differences in left ventricular ejection fraction for all included studies, comparing ‘ablate and pace’ (*n* = 949; 77.3% with cardiac resynchronization therapy) vs. pharmacological therapy (*n* = 1530). MD, mean difference; CRT, cardiac resynchronization therapy; LVEF, left ventricular ejection fraction.

A total of 2479 AF patients were included across 16 studies reporting follow-up LVEF, with 949 (38.3%) in the ‘ablate and pace’ group—734 (77.3%) of whom received CRT—and 1530 (61.7%) in the pharmacological therapy group. Of these studies, three were RCTs including 208 patients, and 13 were observational studies comprising 2271 patients. Overall, ‘ablate and pace’ was associated with a non-significant improvement in LVEF compared with pharmacological therapy (MD, 1.1; 95% CI, −1.6–3.8; *P* = 0.39; *I*² = 72%; *Figure 4*). In observational studies, there was a trend towards greater LVEF improvement with ‘ablate and pace’, although this difference did not reach statistical significance. In contrast, the three RCTs reported a neutral difference in LVEF between treatment strategies.

**Figure 5:**
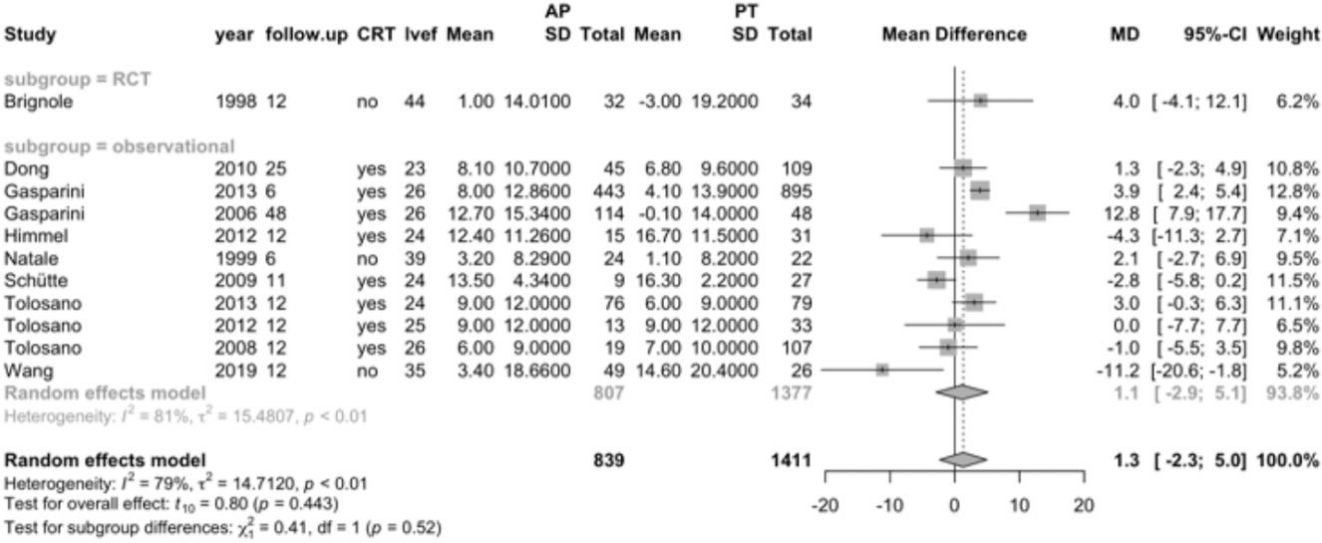
Forest plot of mean differences in left ventricular ejection fraction for the heart failure population, comparing ‘ablate and pace’ (*n* = 839) vs. pharmacological therapy (*n* = 1411). RCT, randomized controlled trial; CRT, cardiac resynchronization therapy; LVEF, left ventricular ejection fraction; follow-up, months; MD, mean difference; AP, ablate and pace; PT, pharmacological therapy.

A total of 2250 patients with AF and heart failure were identified from 11 studies reporting follow-up LVEF values, with mean follow-up durations ranging from 6 to 48 months. In observational studies, the LVEF difference between the ‘ablate and pace’ group (*n* = 807) and the pharmacological therapy group (*n* = 1377) was not statistically significant (MD, 1.1; 95% CI, −2.9–5.1). One RCT compared LVEF between patients treated with ‘ablate and pace’ (*n* = 32) and pharmacological therapy (*n* = 34), also reporting no significant difference (MD, 4.0; 95% CI, −4.1–12.1; *Figure 5*).

**Figure 6:**
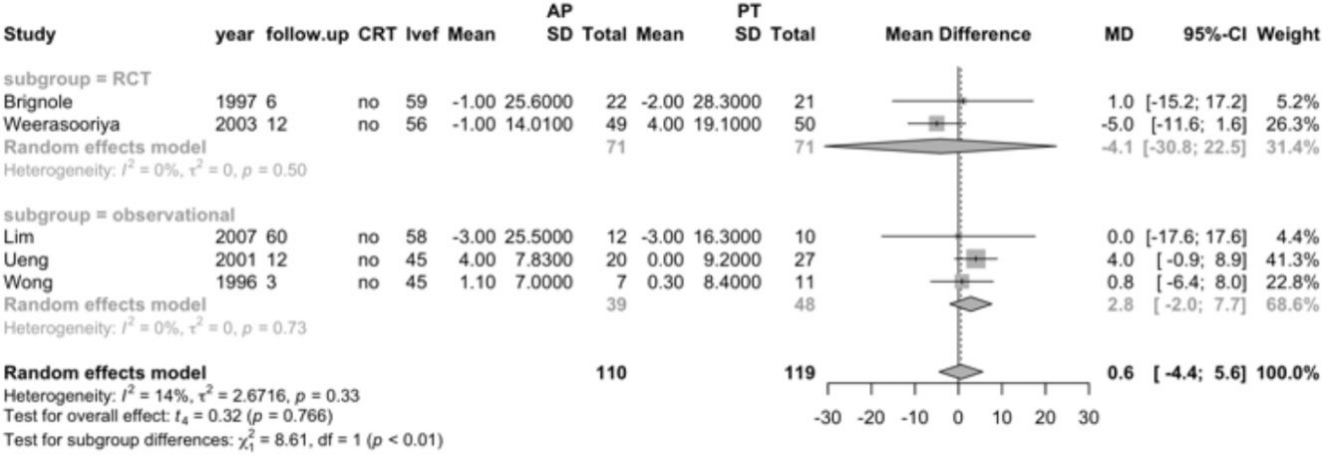
Forest plot of mean differences in left ventricular ejection fraction for the population without heart failure, comparing ‘ablate and pace’ (*n* = 110) vs. pharmacological therapy (*n* = 119). RCT, randomized controlled trial; CRT, cardiac resynchronization therapy; LVEF, left ventricular ejection fraction; follow-up, months; MD, mean difference; AP, ablate and pace; PT, pharmacological therapy.

An additional 229 AF patients without heart failure were included across five studies. In three observational studies, LVEF was similar between the ‘ablate and pace’ group (*n* = 39) and the pharmacological therapy group (*n* = 48) (MD, 2.8; 95% CI, −2.0–7.7). Likewise, two RCTs comparing ‘ablate and pace’ (*n* = 71) to pharmacological therapy (*n* = 71) showed no significant difference in LVEF (MD, −4.1; 95% CI, −30.8–22.5; *Figure 6*).

#### Sensitivity analysis and risk of bias

The Supplementary material contains the sensitivity analyses including temporal analyses of the primary outcomes and a risk of bias assessment.

## Discussion

**‘**Ablate and pace’ is associated with reduced mortality compared with pharmacological therapy in patients with heart failure and predominantly permanent AF, with this association confirmed in sensitivity analyses limited to follow-up periods exceeding 12 months. The results were largely based on observational studies (*n* = 8), which corroborated the few RCTs (*n* = 2) on the topic. In the same comparison of ‘ablate and pace’ with pharmacological therapy, the LVEF was neutral at follow-up and was independent of heart failure status.

### Mortality

Heart failure patients with predominantly permanent AF experience a mortality reduction with the ‘ablate and pace’ strategy compared to pharmacological therapy, particularly beyond 12 months of follow-up. This is supported by one of two RCTs by Brignole *et al.*, with key differences between the studies being the pacing modality—CRT vs. right ventricular pace (RVP)—and sample size (APAF 2021: *n* = 133 vs. 1998 RCT: *n* = 66).^2,8^ We hypothesize that full-time rate control and rhythm regularization through ‘ablate and pace’ may slow heart failure progression by attenuating renin–angiotensin–aldosterone system activation and enhancing myocardial metabolism.^30,31^ This is supported by findings from the RATE-HF RCT, where improved rate control with digoxin in AF patients was associated with significant N-terminal pro–B-type natriuretic peptide (NT-proBNP) reduction, a biomarker that independently predicts reduced mortality.^32,33^

In contrast, among AF patients without heart failure—none of whom received CRT, as these studies (from 2001, 2003, 2007, and 2011) predated clear guideline recommendations for CRT in patients with high pacing burden and preserved LVEF—no mortality difference was observed between ‘ablate and pace’ and drug treatment. Notably, guidelines first addressed CRT for high pacing burden and reduced LVEF in 2012, and the 2022 American Heart Association/American College of Cardiology/Heart Failure Society of America (AHA/ACC/HFSA) guidelines now extend this to select patients with LVEF > 40%.^34,35^ The low baseline mortality risk and limited events likely explain the lack of treatment effect; larger, longer trials would be needed to confirm any benefit in this population.^36^

This suggests that AF-related mortality in heart failure patients may be mediated by uncontrolled rate and irregular rhythm contributing to heart failure progression—a mechanism less relevant in patients without heart failure, at least in the short term. Nevertheless, in the long-term management of AF without heart failure, ‘ablate and pace’ may be indicated in specific scenarios: (i) failure or contraindication of PVI, (ii) prevention or reversal of tachycardia-induced cardiomyopathy, and (iii) persistent symptomatic AF with uncontrolled ventricular rate despite optimal medical therapy.

### Left ventricular ejection fraction

We found LVEF to be neutral in the comparison of ‘ablate and pace’ with pharmacological therapy and independent of heart failure.

In AF patients, we expect medical interventions to have a neutral effect on the LVEF unless linked with acute conditions. Examples of acute conditions with potentially reversible LVEF declines are tachycardia-induced cardiomyopathy, Takotsubo syndrome, and acute myocardial infarction.^37,38^ In our meta-analysis, patients suffered mainly from permanent AF where pharmacological therapy failed to achieve optimal rate control and rhythm regularization, but none of the included studies were linked to acute conditions.

In AF patients without heart failure, we also expected a neutral LVEF outcome due to either mildly reduced or steady LVEF at baseline.

### ‘Ablate and pace’

Our findings suggest that the combination of ‘ablate and pace’ therapy may reduce mortality in patients with heart failure and AF by providing consistent rate control and rhythm regularization. This aligns with evidence from animal studies dating back to the 1960s, where swine and canine models demonstrated that sustained rapid pacing induces tachycardia-mediated cardiomyopathy, characterized by reduced LVEF and myocardial remodelling.^39,40^ These results support the rationale for rate control strategies in this population. Given this pathophysiology, the modest or absent improvement in LVEF observed in our study is not unexpected, as the primary benefit of pacing after ablation may be through haemodynamic optimization rather than direct myocardial recovery.

Historically, CRT responses have been shown in heart failure patients with sinus rhythm and BBB, as demonstrated in Cleland *et al.*’s 2014 meta-analysis.^39^ However, RCTs have demonstrated the superiority of CRT in heart failure patients with AF, including the BLOCK (which included 53% AF patients), the Budapest, and APAF (2011) trials.^41–43^

Based on our analysis, we conclude that LVEF may not be a reliable marker for the success of ‘ablate and pace’ therapy in patients with heart failure and AF, as no significant improvement in LVEF was observed.

### Other meta-analyses

In a meta-analysis from 2012, Stavrakis *et al.*^44^ compared ‘ablate and CRT’ with ‘ablate and RVP’ from five RCTs, which enrolled symptomatic AF patients (<50% with BBB). They found mortality neutral in the comparison of the two interventions and reported a significant mean LVEF difference of 2.0 favouring CRT. The LVEF improvement was weighted 60% on the APAF RCT from 2011 in their meta-analysis.^43^ The APAF RCT itself was not able to demonstrate a significant LVEF difference between CRT and RVP. We suggest that a CRT response remains unlikely in AF, since a marginal mean LVEF improvement and a neutral mortality are insufficient to consider a CRT response. An earlier meta-analysis on observational studies by Wood *et al.*^45^ reported significant improvements in all their outcomes after ‘ablate and pace’, including quality of life and the LVEF, with the exception of fractional shortening. The cross-over design of their included studies meant that patients acted as their own controls, thereby increasing the risk of a placebo effect.

A meta-analysis from 2014 by Yin *et al.*,^46^ which included only patients with permanent AF, LVEF < 35%, and BBB, demonstrated a significant 37% reduction in mortality risk in the ‘ablate and pace’ group compared with pharmacological therapy alone; all patients received CRT. They concluded that ‘ablate and pace’ significantly reduced the risk of CRT non-response in patients with inadequate biventricular pacing (≤90%), whereas in those with sufficient pacing (>90%), ablation conferred no additional benefit. Thus, suboptimal pacing identified a subgroup in whom ablation was particularly beneficial to achieve effective CRT.

### Implications

In leading guidelines, ‘ablate and pace’ is indicated for symptomatic relief in medically refractory AF independent of the LVEF.^1^ Our meta-analysis suggests that ‘ablate and pace’ reduces mortality in AF patients with heart failure through full-time rate control and rhythm regularization. Cardiac resynchronization therapy remains the preferred PM device due to the established literature on PM-induced cardiomyopathy in patients with right ventricular pacing.^47^

*A priori*, PVI would be expected to be superior to ‘ablate and pace’ in AF patients due to improved chronotropic function and atrioventricular synchrony, where ‘ablate and pace’ affords improved chronotropic function at the cost of PM dependency.

Both the CASTLE RCTs bolstered PVI superiority: PVI improved survival and heart function as compared with pharmacological therapy in AF patients with heart failure.^48,49^

The PABA-HF RCT demonstrated significant superiority of PVI vs. ‘ablate and CRT-D’ in 81 AF patients with heart failure in terms of mortality.^50^ Selecting the right approach −rate or rhythm control—is crucial. Including asymptomatic patients with permanent AF and moderate to severe heart failure in future RCTs could provide valuable insights for investigating ‘ablate and pace’ therapies. They may compare His-bundle pacing with CRT to assess left ventricular dyssynchrony improvement.^51^ Finally, AF patients with implantable cardioverter-defibrillator (ICD) and medically refractory AF may benefit from ‘ablate and CRT upgrade’ if they suffered from inappropriate ICD shocks in the past.^52^

### Limitations

The take-home message of mortality reduction more than 12 months after ‘ablate and pace’ in heart failure patients with predominantly permanent AF is limited by the low number of RCTs in our meta-analysis. Thus, the European Society of Cardiology guidelines’ recent Class 2A recommendation and Level of Evidence B stands in line with our results.^53^ Here, observational study results may add unknown confounder bias to the outcomes and therefore false signalling. Also, patient selection bias is a risk in observational studies. In our meta-analysis, we acknowledge that more AF symptoms possibly favoured ‘ablate and pace’ and even cross-over in the RCTs. This, unfortunately, leaves the question open in terms of the mortality reduction in less symptomatic patients with heart failure and permanent AF, who make up the larger part of the AF patient population with heart failure by far. Our meta-analysis therefore reveals statistical associations, which so far have been tested for causality in four RCTs. Two of the RCTs corroborated our observational findings: ‘ablate and pace’ decreases mortality as compared with pharmacological therapy in heart failure patients with permanent AF. Another limitation of our meta-analysis is the use of an LVEF threshold of <40% to define the heart failure population, which may have led to misclassification in patients with transiently reduced LVEF. However, we consider this risk minimal, as patients with AF and moderately reduced LVEF typically have elevated NT-proBNP levels, supporting the presence of true systolic heart failure.^54^

It is also acknowledged that AF symptoms may have overlapped with heart failure symptoms in the included studies. From a clinical perspective, the optimal timing of ‘ablate and pace’ in patients with permanent AF and heart failure remains to be determined. We could neither establish a separate impact of few paroxysmal AF patients on mortality nor identify specific antiarrhythmic pharmacological therapy in a sound way. This contributes uncertainty to their impact on the outcomes. The Castle HF and HTx RCTs enrolled patients with both permanent and paroxysmal AF and made no constraints to antiarrhythmic pharmacological therapy due to the missing evidence of their distinct treatment effects. We suggest that symptomatic relief and reduced mortality can be achieved in both permanent and paroxysmal AF patients with heart failure through ‘ablate and pace’ but that it should be targeted to elderly patients where PVI is assessed as unfeasible.

Despite missing data being a caveat of our meta-analysis, sensitivity analyses corroborated the primary analyses. Even the imputed standard deviations of the LVEF MD provided robust results. In addition, the observational studies and the few RCTs included found similar pooled results in their assessment of ‘ablate and pace’ compared with pharmacological therapy.

The moderate to high heterogeneity observed was mainly attributed to the inclusion of observational studies, which were more prevalent than RCTs. Additionally, semiquantitative echocardiographic measurements of LVEF contributed to substantial heterogeneity. Given that LVEF plays a key role in heart failure diagnosis, it could influence the magnitude of mortality outcomes. To minimize bias, random effects meta-analyses were performed, and interim analyses showed no significant differences in primary outcomes between study designs.

## Conclusions

**‘**Ablate and pace’ reduced mortality in patients as compared with pharmacological therapy, which was supported by statistical associations in observational studies. One RCT out of two corroborated the finding. Thus, more confirmatory RCTs are welcome. Supplementary material is available online.

## Supplementary Material

oeag020_Supplementary_Data

## Data Availability

The data underlying this article are derived from published RCTs and observational studies identified through systematic searches of electronic bibliographic databases. No new primary data were generated. All source data are available in the cited articles. The dataset generated during the current study is available from the corresponding author upon reasonable request.
