# Supplementary material for: ‘Ablate and pace’ reduces mortality in heart failure patients with atrial fibrillation: an updated meta-analysis
Source: Eur Heart J Open. 2026 Feb 18;6(2):oeag020. doi: 10.1093/ehjopen/oeag020 (PMC12988461; doi:10.1093/ehjopen/oeag020)
Supplement: oeag020_Supplementary_Data [file oeag020_supplementary_data.docx]

# Supplementary materials

Authors: Christian Lewinter^1,2,3^, John GF Cleland^2^, Eslem Sögütlü^3^, Torsten Holm Nielsen^4-5^, Hannes Hagström^3^,

Lars Køber^6^, Martin LeWinter^7^, Robert Edfors^3^, Cecilia Linde^1,3^, Frieder Braunschweig^1,3^.

(1)Karolinska University Hospital, Heart Centre, Stockholm, Sweden; (2) University of Glasgow, School of cardiovascular and metabolic health, United kingdom; (3) The Karolinska institute, Stockholm, Sweden (4) Danish Medicines Agency, Denmark; (5) Department of Haematology, Zealand University Hospital(6) Rigshospitalet, Copenhagen University Hospital, Heart Centre; Denmark; (7) White River Junction Veterans Affairs Medical Center, Division of Cardiovascular medicine,Vermont, United states of America.

# Supplementary figures

# Figure S0: Distribution of HF, without HF, CRT and study design……………………………………………………… p.2

# Sensitivity analysis……………………………………………………………………………………………................. p.3

# Sensitivity analysis results………………………………………………………………………………………………..p.3

# Figure S1: Time frames ≤ 12 months and > 12 months of mortality (all studies)………...……………………………... p.4

# Figure S2: Time frames ≤ 12 months and > 12 months of mortality (HF)……………………………………................. p.4

# Figure S3: Time frames ≤ 12 months and > 12 months of mortality (without HF)….....………………………............... p.5

# Figure S4: Time frames ≤ 12 months and > 12 months of LVEF (all)…………………………………………………... p.5

# Figure S5: Time frames ≤ 12 months and > 12 months of LVEF (HF)………………………………………………….. p.6

# Figure S6: Time frames ≤ 12 months and > 12 months of LVEF (without HF)………..………………………………... p.6

# Figure S7: Replace by the mean for LVEF outcomes……………………………………………………………………. p.7

# Figure S8: Follow-up LVEF……………………………………………………………………………………………... p.7

# Figure S9: Per protocol for the LVEF……...…………………………………………………………………………….. p.8

# Supplementary tables

# Table 1: distribution of pacemaker mode………………………………………………………………………………… p.9

# Risk of bias methods………………....……...…………………………………………………………………………… p.10

# Risk of bias………..………………....……...…………………………………………………………………………… p.11

# Table T2: risk of bias for observational studies……………………………………………………………….................. p.12

# Table T3: risk of bias for RCTs………………………………………………………………………………................... p.13

# Charts

# Prisma flow charts……………………………………………………………………………………………................... p.14

Figure S0: Distribution of HF, without HF, CRT and study design

Sensitivity analysis

For the LVEF outcome, imputations were calculated for missing SDs of the LVEF MD between groups through 4 different methods. First, a correlation coefficient according to Abrams et al. was obtained from studies with complete data of LVEF MD and associated standard deviations (1). Then, a single weighted coefficient was imputed and weighted as the average from the distinct study coefficients calculated from complete datasets. The final coefficient averaged both the ‘ablate and pace’ and pharmacological therapy groups.

Second, missing SDs of the MDs were substituted from studies with complete data. An average value of the reported SDs was weighted by study participants.

Third, an alternative analysis estimated the MD of the follow-up LVEFs between the ‘ablate and pace’ and pharmacological therapy groups.

Fourth, as a per-protocol analysis of the LVEF MD, only studies with full datasets were applied.

For both the mortality and LVEF outcomes, we distinguished between observational studies, RCTs, and participants with and without heart failure. A timeframe distinction of > 12 months was undertaken for all the

analyses.

1. Abrams KR, Gillies CL, Lambert PC. Meta-analysis of heterogeneously reported trials assessing change from baseline. Statist Med. 2005 Dec 30;24(24):3823–44.

Sensitivity analysis results

When stratifying studies by duration of follow-up, the mortality endpoint was significantly reduced only in studies with follow-up longer than 12 months (Figure S1–S4). In comparison, differentiation of study duration ≤ 12 months versus > 12 months did not affect the LVEF endpoint (Figure S4–S6).

In the comparison of ‘ablate and pace’ with pharmacological therapy, replacement of missing SDs of the LVEF MD with an average value from studies with fully reported SDs resulted in a non-significant average LVEF MD for all the studies, as well as in the differentiation of RCTs and observational studies (**Figure S7**).

Using the follow-up LVEF and associated SDs as the outcomes, instead of the LVEF MD, yielded similar results (MD, 1.7; 95% CI, -1.0-4.4; P= 0.19, I^2^= 83%; Figure S8). The same neutrality between ‘ablate and pace’ pharmacological therapy was found when differentiating between the observational and RCT subgroups

A similar non-significant average LVEF MD (MD, 1.3; 95% CI, -0.7- 3.4; P= 0.15, I^2^= 0%) was found when only studies with full reports of SDs of the MDs were included (Figure S9).

Figure S1: Time frames ≤ 12 months and > 12 months of mortality (all studies)

For ≤ 12 months: Forest plot of mortality in the comparison of ‘ablate and pace’ (n=165) with pharmacological therapy (n=170). For > 12 months: ‘ablate and pace’ (n=1444) and pharmacological therapy (n=1886). CRT: Cardiac resynchronisation therapy. Follow.up: months. AP: ‘ablate and pace. PT: Pharmacological therapy.

Figure S2: Time frames ≤ 12 months and > 12 months of mortality (HF patients)


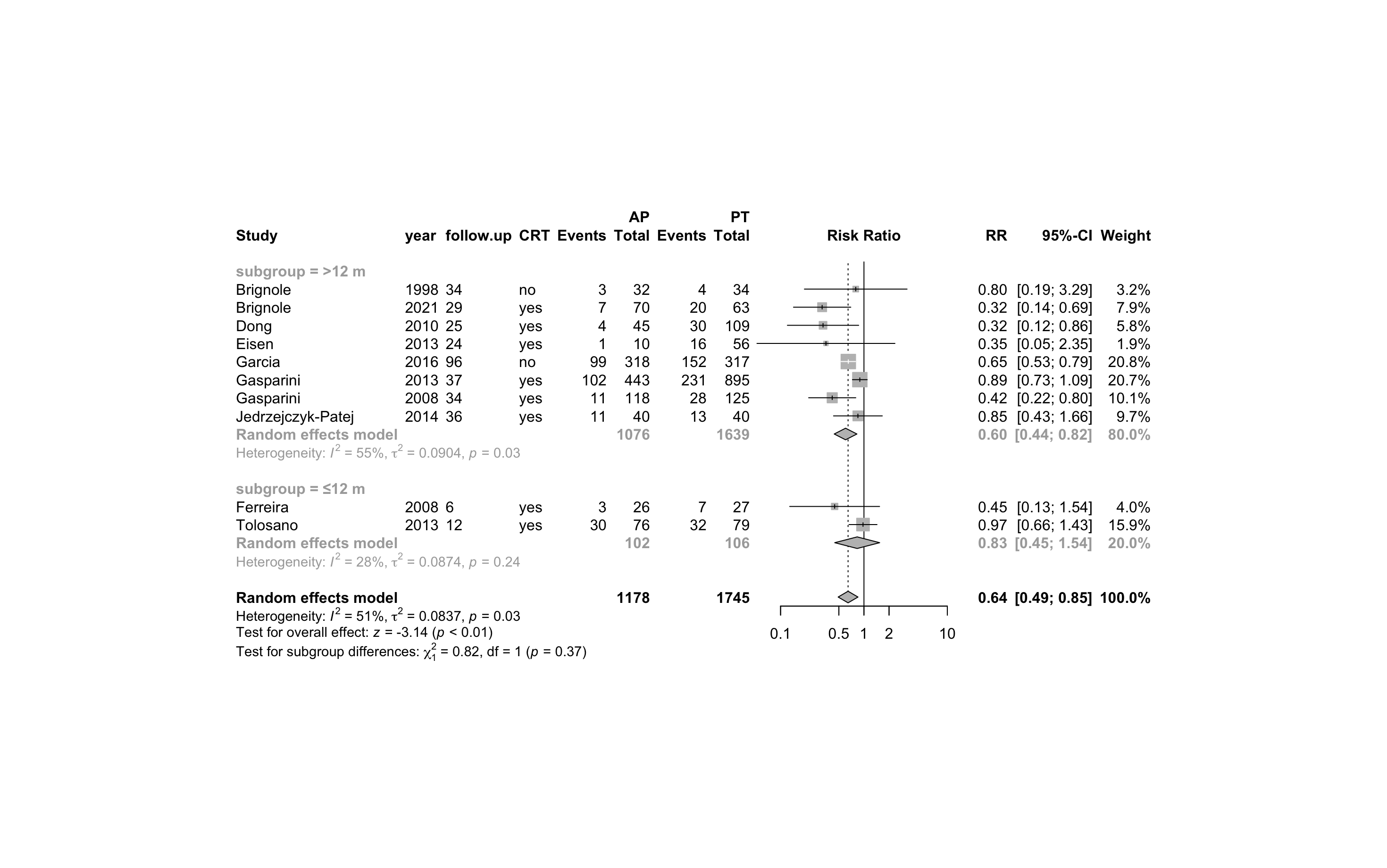
For ≤ 12 months: Forest plot of mortality in the comparison of ‘ablate and pace’ (n=102) with pharmacological therapy (n=106). For > 12 months: ‘ablate and pace’ (n=1076) and pharmacological therapy (n=1639). CRT: Cardiac resynchronisation therapy. Follow.up: months. AP: ‘ablate and pace. PT: Pharmacological therapy.

Figure S3: Time frames ≤ 12 months and > 12 months of morality (without HF patients)

For ≤ 12 months: Forest plot of mortality in the comparison of ‘ablate and pace’ (n=63) with pharmacological therapy (n=64). For > 12 months: ‘ablate and pace’ (n=368) and pharmacological therapy (n=247). CRT: Cardiac resynchronisation therapy. Follow.up: months. AP: ‘ablate and pace. PT: Pharmacological therapy.

Figure S4: Time frames ≤ 12 months and > 12 months of LVEF (all)

For ≤ 12 months: Forest plot of LVEF in the comparison of ‘ablate and pace’ (n=778) with pharmacological therapy (n=1363). For > 12 months: ‘ablate and pace’ (n=171) and pharmacological therapy (n=167). CRT: Cardiac resynchronisation therapy. lvef: Left ventricular ejection fraction. Follow.up: months. AP: ‘ablate and pace. PT: Pharmacological therapy.

Figure S5: Time frames ≤ 12 months and > 12 months of LVEF (HF patients)

For ≤ 12 months: Forest plot of LVEF in the comparison of ‘ablate and pace’ (n=680) with pharmacological therapy (n=1254). For > 12 months: ‘ablate and pace’ (n=159) and pharmacological therapy (n=157). CRT: Cardiac resynchronisation therapy. lvef: Left ventricular ejection fraction. Follow.up: months. AP: ‘ablate and pace. PT: Pharmacological therapy.

Figure S6: Time frames ≤ 12 months and > 12 months of LVEF (without HF patients)

For ≤ 12 months: Forest plot of LVEF in the comparison of ‘ablate and pace’ (n=98) with pharmacological therapy (n=109). For > 12 months: AVJA (n=12,) and pharmacological therapy (n=10).

CRT: Cardiac resynchronisation therapy. lvef: Left ventricular ejection fraction. Follow.up: months. AP: ‘ablate and pace. PT: Pharmacological therapy.

Figure S7: Replace by the mean for LVEF outcomes (all patients)

Replacement by the mean standard deviation: Forest plot of LVEF mean differences comparing ‘ablate and pace’ (n=949) with pharmacological therapy (n=1530). RCT: randomised controlled trials. CRT: Cardiac resynchronisation therapy. Lvef: Left ventricular ejection fraction. Follow.up: months. AP: ‘ablate and pace. PT: Pharmacological therapy.

Figure S8: Follow-up LVEF (all patients)


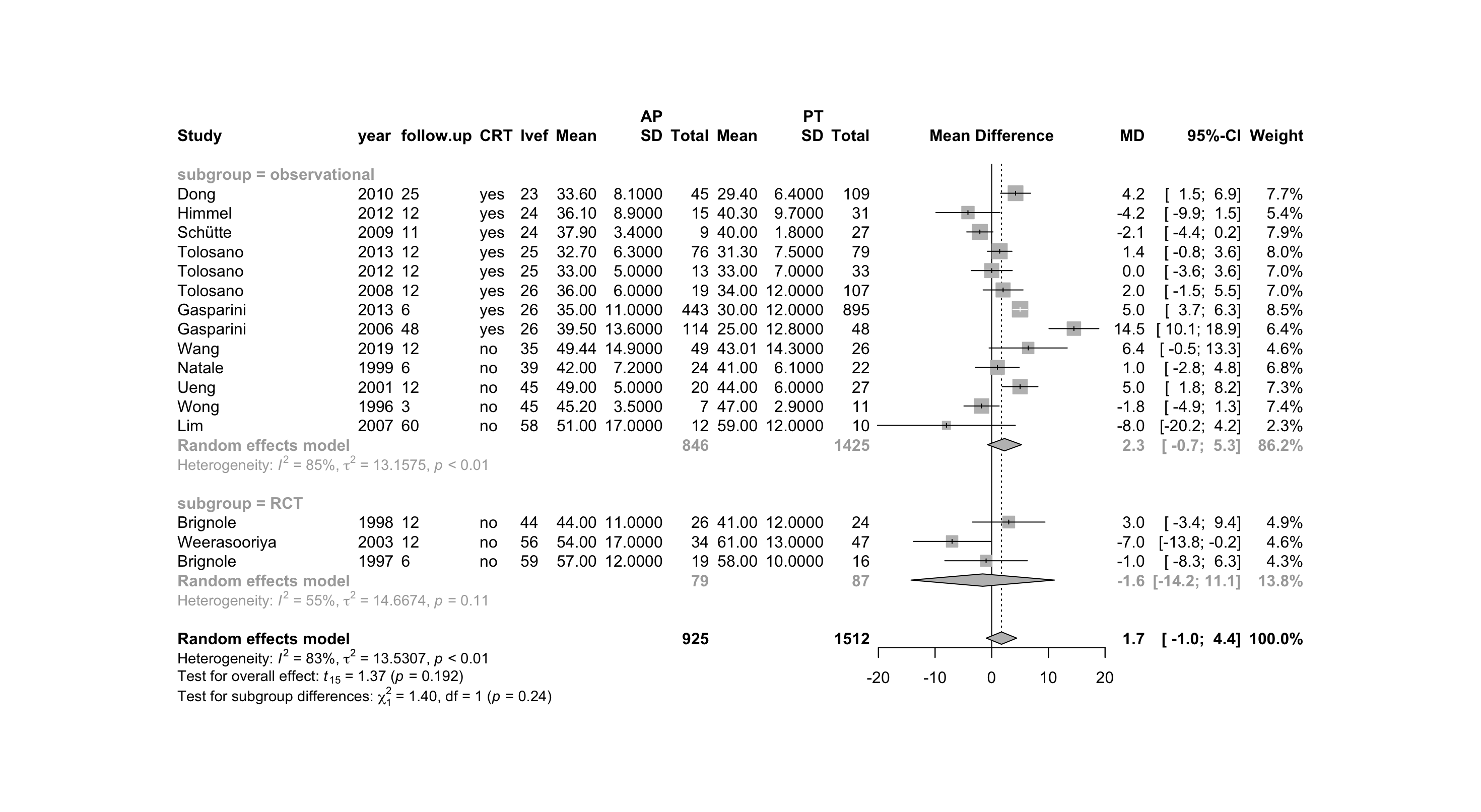
LVEF mean difference at follow-up: Forest plot of LVEF in the comparison of ‘ablate and pace’ (n=925) with pharmacological therapy (n=1401). RCT: randomised controlled trials. CRT: Cardiac resynchronisation therapy. Lvef: Left ventricular ejection fraction. Follow.up: months. AP: ‘ablate and pace. PT: Pharmacological therapy.

Figure S9: Per protocol (all patients)

Per protocol analysis: Forest plot of LVEF mean differences comparing ‘ablate and pace’ (n=160) with pharmacological therapy (n=339). RCT: randomised controlled trials. CRT: Cardiac resynchronisation therapy. Lvef: Left ventricular ejection fraction. Follow.up: months. AP: ‘ablate and pace. PT: Pharmacological therapy.

Table S1: distribution of pacemaker mode

| Study | Year | CRT(%) | VVI(%) | DDD(R)(%) |
| --- | --- | --- | --- | --- |
| Brignole | 1997 | 0 | 0 | 100 |
| Brignole | 1998 | 0 | 100 | 0 |
| Brignole | 2021 | 100 | 0 | 0 |
| Dong | 2010 | 100 | 0 | 0 |
| Eisen | 2013 | 100 | 37 | 0 |
| Ferreira* | 2008 | 77 | 23 | 0 |
| Garcia* | 2016 | 37 | 63 | 0 |
| Gasparini | 2006 | 100 | 0 | 0 |
| Gasparini | 2008 | 100 | 0 | 0 |
| Gasparini | 2013 | 100 | 0 | 0 |
| Himmel | 2012 | 100 | 0 | 0 |
| Jedrzejczyk-Patej | 2014 | 100 | 0 | 0 |
| Lim | 2007 | 0 | 100 | 0 |
| Nagamoto | 2011 | 0 | 0 | 100 |
| Natale | 1999 | 0 | 100 | 0 |
| Ozcan | 2001 | 0 | 55 | 45 |
| Schütte | 2009 | 100 | 0 | 0 |
| Tolosano | 2008 | 100 | 0 | 0 |
| Tolosano | 2012 | 100 | 0 | 0 |
| Tolosano | 2013 | 100 | 0 | 0 |
| Ueng | 2001 | 0 | 100 | 0 |
| Wang* | 2019 | 0 | 0 | 0 |
| Weerasooriya | 2003 | 0 | 100 | 0 |
| Wong | 1996 | 0 | 100 | 0 |

*The “ablate and pace” group had 100% his bundle pacing.

CRT= Cardiac resynchronisation therapy, VVI= right ventricular single-chamber pacing,

DDD(R)= dual chamber pacing with rate adaptation.

Risk of bias methods

Adequate sequence generation, allocation concealment, blinding, incomplete outcomes, and selective reporting were assessed according to the Cochranes Collaboration’s tool for risk of bias in randomised trials (1). For observational studies, the ROBINS-I tool addressed confounders, selection bias, information bias and reporting bias (2).

1. Higgins JPT, Thomas J, Chandler J, Cumpston M, Li T, Page MJ, Welch VA (editors). Cochrane Handbook for Systematic Reviews of Interventions version 6.0 (updated July 2019). Cochrane, 2019. Available from [www.training.cochrane.org/handbook](http://www.training.cochrane.org/handbook).
2. Sterne JA, Hernán MA, Reeves BC, Savović J, Berkman ND, Viswanathan M, et al. ROBINS-I: a tool for assessing risk of bias in non-randomised studies of interventions. BMJ. 2016 Oct 12;i4919.

Risk of bias

In RCTs we found concerns in the randomisation process. This was due to lack of description of the randomisation. Missing data was assessed as high risk of bias in all the studies. Patients not completing the randomised trials varied between 5 to 18%. Bias in the measurements of the LVEF gave rise to concern due to the semiquantitative nature of echocardiography, which was the preferred method. We found it unlikely that the interventions deviated due to randomised designs. Nor did we find selective reporting of the outcomes.

The overall bias in the observational studies was moderate. Confounding bias at baseline was unavoidable due to patient selection of the ‘ablate and pace’ group. This was in general determined by unsuccessful pharmacological therapy of tachycardia or symptoms during atrial fibrillation. We found a moderate risk of bias in the interventions since many studies were based on multisided data acquisition. Missing data was low in most of the studies but difficult to assess since the patient populations were likely to be selected. Selective reporting of data was assessed to be more prevalent in observational studies as compared with RCTs. This was explained by the retrospective nature of the data analyses, which made it difficult to decide if the outcomes were pre-specified before the analysis. Despite this difficulty, a large part of the outcomes was neutral in the comparison of ‘ablate and pace’ with pharmacological therapy.

Table T2: risk of bias for observational studies


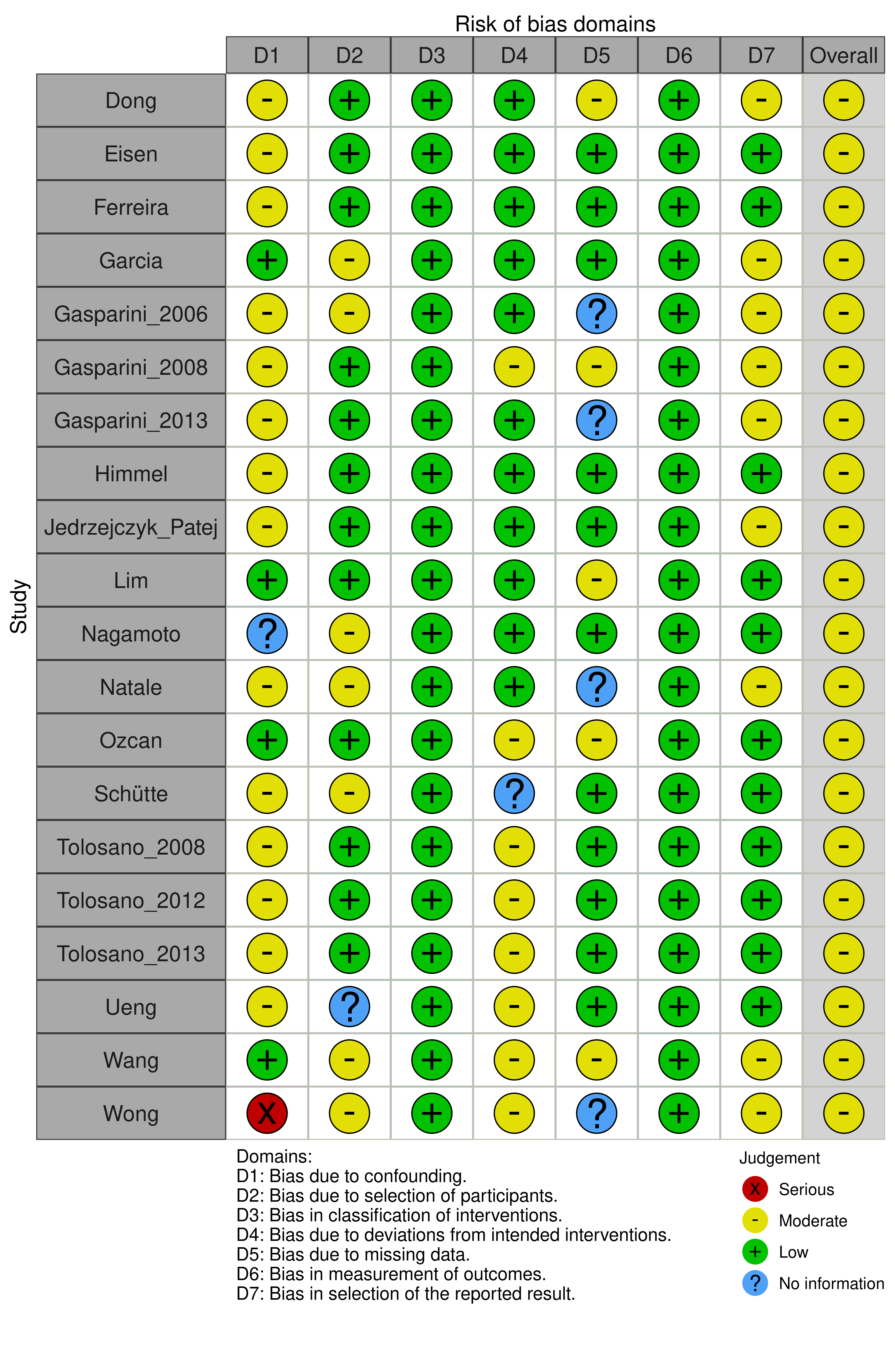
Table T3: risk of bias for RCTs


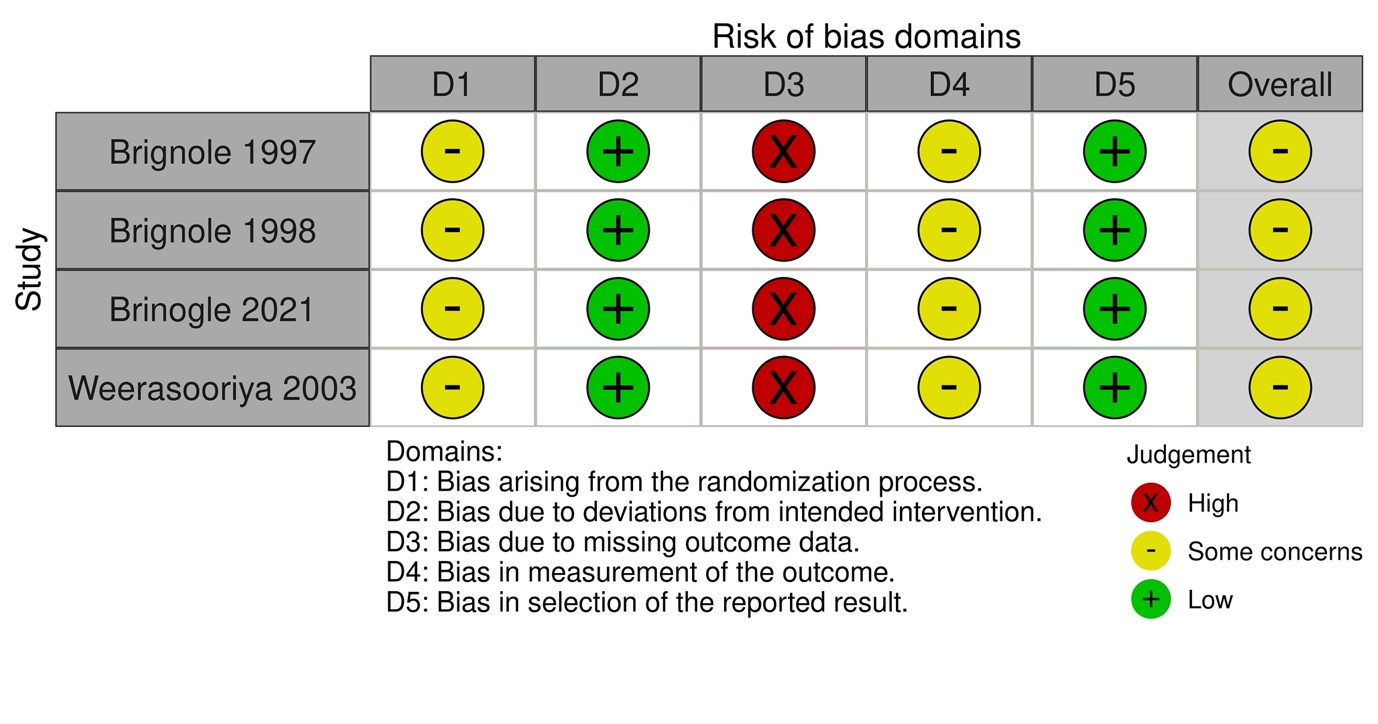


Prisma flow charts

Identification of studies via databases and registers

Records removed *before screening*:

Duplicate records removed

(n = 806 )

Records marked as ineligible by automation tools (n = 4)

Records removed for other reasons (n = 0)

Records identified from:

PubMed (n =2803)

Embase (n = 678 )

CENTRAL(n =356 )

Identification

Records screened

(n =3027)

Records excluded

(n =2920)

Reports sought for retrieval

(n = 107)

Reports not retrieved

(n = 0 )

Screening

Reports assessed for eligibility

(n = 107)

Reports excluded (n =83):

Reason 1: study design

Reason 2: outcome

Studies included in review

(n =24)

Included

*From:*  Page MJ, McKenzie JE, Bossuyt PM, Boutron I, Hoffmann TC, Mulrow CD, et al. The PRISMA 2020 statement: an updated guideline for reporting systematic reviews. BMJ 2021;372:n71. doi: 10.1136/bmj.n71
